# Identification of CmbHLH Transcription Factor Family and Excavation of *CmbHLHs* Resistant to Necrotrophic Fungus *Alternaria* in *Chrysanthemum*

**DOI:** 10.3390/genes14020275

**Published:** 2023-01-20

**Authors:** Yifeng Ding, Xiaomeng Wang, Dandan Wang, Liwei Jiang, Jing Xie, Tianle Wang, Lingyu Song, Xiting Zhao

**Affiliations:** 1 Department of Biological Sciences, College of Life Sciences, Henan Normal University, Xinxiang 453007, China; 2 Engineering Technology Research Center of Nursing and Utilization of Genuine Chinese Crude Drugs in Henan Province, Xinxiang 453007, China; 3 Engineering Laboratory of Biotechnology for Green Medicinal Plant of Henan Province, Xinxiang 453007, China

**Keywords:** *Chrysanthemum morifolium* Ramat., bHLH transcription factor, *CmbHLHs*, resistance, necrotrophic fungus

## Abstract

*Chrysanthemum morifolium* Ramat. ‘Huaihuang’ is a traditional Chinese medicinal plant. However, a black spot disease caused by *Alternaria* sp., a typical necrotrophic fungus, has a serious damaging influence on the field growth, yield, and quality of the plant. ‘Huaiju 2^#^’ being bred from ‘Huaihuang’, shows resistance to *Alternaria* sp. bHLH transcription factor has been widely studied because of their functions in growth development, signal transduction, and abiotic stress. However, the function of bHLH in biotic stress has rarely been studied. To characterize the resistance genes, the CmbHLH family was surveyed in ‘Huaiju 2^#^’. On the basis of the transcriptome database of ‘Huaiju 2^#^’ after *Alternaria* sp. inoculation, with the aid of the *Chrysanthemum* genome database, 71 *CmbHLH* genes were identified and divided into 17 subfamilies. Most (64.8%) of the CmbHLH proteins were rich in negatively charged amino acids. CmbHLH proteins are generally hydrophilic proteins with a high aliphatic amino acid content. Among the 71 CmbHLH proteins, five *CmbHLHs* were significantly upregulated by *Alternaria* sp. infection, and the expression of *CmbHLH18* was the most significant. Furthermore, heterologous overexpression of *CmbHLH18* could improve the resistance of *Arabidopsis thaliana* to necrotrophic fungus *Alternaria brassicicola* by enhancing callose deposition, preventing spores from entering leaves, reducing ROS accumulation, increasing the activities of antioxidant enzymes and defense enzymes, and promoting their gene expression levels. These results indicate that the five *CmbHLHs*, especially *CmbHLH18*, may be considered candidate genes for resistance to necrotrophic fungus. These findings not only increase our understanding of the role *CmbHLHs* play in biotic stress but also provide a basis by using *CmbHLHs* to breed a new variety of *Chrysanthemum* with high resistance to necrotrophic fungus.

## 1. Introduction

*Chrysanthemum morifolium* Ramat., a perennial herb with a wide variety of flower types, has been widely used in cut flowers, potting, and garden greening [1]. *C. morifolium* ‘Huaijuhua’ produced mainly in Jiaozuo city of Henan province, China, is one of the Four Famous Huai Herbals; it has been cultivated for more than three thousand years in China. Its annual planting area is approximately 800 hm^2^ with an output value of one hundred and ninety million Yuan [2]. ‘Huaijuhua’ is rich in three kinds of medicinal active ingredients, namely luteoloside, chlorogenic acids, and 3, 5-dicaffeoyl-quinic acid [3]. As a traditional medicinal Chinese *Chrysanthemum*, besides its medicinal value, it can also be used as ornamental and drinking tea [3]. However, ‘Huaijuhua’ is subjected to year-round propagation through cuttings and division propagation, so it is susceptible to a variety of pathogens during the growth stage. Our previous investigation showed that a black spot disease caused by *Alternaria* sp., a typical necrotrophic fungus, has a serious negative impact on the field growth, yield, and quality of ‘Huaijuhua’ [4]. The incidence of black spot was 30% to 80%, even up to 100% in the field planting of ‘Huaijuhua’ [5]. After six years of selection and cultivation, a new resistant cultivar ‘Huaiju 2^#^’ has been bred. This makes it possible to characterize potential resistance genes from ‘Huaiju 2^#^’.

Recently, bHLH (basic helix-loop-helix) transcription factors (TFs) have been widely studied because of their functions in signal transduction, biosynthesis, response to various stresses, and growth regulation of plants [6]. bHLH TFs are named for having a highly conserved bHLH domain. The bHLH domain of this family contains about 50–60 amino acid residues with the N-terminal basic region (b) and the C-terminal helix-loop-helix (HLH) [6]. The HLH region can contribute to the formation of homodimers or heterodimers between proteins [7,8]. Leu-23 and Leu-52 are necessary for the formation of dimer complexes of these identified members of the bHLH family [9,10].

Since bHLH proteins were first found in maize [11], they have been successively identified in other plants, including *Arabidopsis*, rice, poplar, potato, cabbage, and so on [12]. However, the bHLH family in *Chrysanthemum* is still unknown. 

Studies have shown that bHLH regulates multiple physiological progresses of plants. It is widely involved in all kinds of signal transduction mediated by plant hormones, such as gibberellic acid (GA), abscisic acid (ABA), and brassinosteroids [10,13,14]. bHLH has also been involved in response to a variety of abiotic stresses, including drought, cold, and salt stress, as well as regulation of iron homeostasis [15,16,17,18]. bHLHs are also related to biological processes, including the biosynthesis of anthocyanins, flavonoids, and ethylene [19,20]. They also take part in the growth regulation of plants, including cell elongation, the growth of fruit and flowers, the development of root hairs and stomata, and senescence of the leaf [21,22,23,24,25]. However, the functions of bHLH in biotic stress are still rarely studied, with a few exceptions in some model plants and crops. For example, heterologous overexpression of *TabHLH060* significantly enhanced *Arabidopsis* sensitivity to the biotrophic pathogen *Pst*. DC3000 [26]. The defense of *Phytophthora nicotiana* of tobacco was associated with 28 NbbHLHs [27], and overexpression of *GhbHLH171* activated the synthesis and signaling pathway of JA and improved the resistance of cotton to the necrotrophic pathogen *V. dahlia* [28].

According to the transcriptome database (Accession number: PRJNA448499) of ‘Huaiju 2^#^’ after *Alternaria* sp. inoculation, using the *Chrysanthemum* genome database (http://www.amwayabrc.com/zh-cn/index.html, accessed on 22 January 2022), CmbHLHs were identified in this study. Their phylogenetic relationships were analyzed. Candidate *CmbHLHs* of resistance to *Alternaria* sp. were confirmed. Furthermore, based on the expression patterns of the *CmbHLHs* under inoculation with *Alternaria* sp., this study also focused on the role of *CmbHLH18* in enhancing the resistance of *Arabidopsis* to the typical necrotrophic fungus, *Alternaria brassicicola*, through heterologous overexpression. This will provide a basis for a deeper understanding of the functions of bHLHs in *Chrysanthemum*.

## 2. Materials and Methods

### 2.1. Plant Materials and Pathogenic Strains

Following the culture method of Zhao et al. [3], both *Chrysanthemum morifolium* ‘Huaiju 2^#^’ (Appreciation of Traditional Chinese Medicine in Henan Province: 2016002) and *Arabidopsis thaliana* (Columbia, Col-0) used in this study were grown in 13 cm diameter pots containing a peat-vermiculite (*v*/*v* = 1:1) mixture in the controlled greenhouse at 20–25 °C, 40–60% relative humidity and under a LD cycle (14 h light/10 h dark), 60 mE·s^−1^ m^−2^ light intensity.

*Alternaria* sp. (Strain: HQJH10092301; GenBank accession number: KF688111) and *A. brassicicola* (Strain: BCHB13101606; GenBank accession number: OP295488) were used as pathogenic strains to inoculate the plants. The plants and fungi are maintained for research purposes at the Engineering Technology Research Center of Nursing and Utilization of Genuine Chinese Crude Drugs in Henan Province, Henan Normal University, Xinxiang, China.

### 2.2. Screening and Physicochemical Properties Analysis of the CmbHLH TF Family

Sequences annotated as the bHLH in the ‘Huaiju 2^#^’ transcriptome database (Accession number: PRJNA448499) were aligned to the *Chrysanthemum* genome database using BLAST on the http://www.amwayabrc.com/zh-cn/index.html (accessed on 22 January 2022). The retrieved candidate genes were checked through the CD-HIT website server (http://weizhongli-lab.org/cd-hit/, accessed on 9 November 2021) to delete redundant sequences [29]. NCBI CDD (https://www.ncbi.nlm.nih.gov/Structure/bwrpsb/bwrpsb.cgi, accessed on 10 November 2021) and SMART (http://smart.embl-heidelberg, accessed on 11 November 2021) were used to remove sequences without conserved bHLH domains, incomplete CDS sequences were also removed, and sequences with typical bHLH domains were selected as putative bHLH TF family members [30,31].

Based on the identified amino acid sequence of the CmbHLH protein, the ExPASY online program (https://www.expasy.org/, accessed on 15 November 2021) was used to predict the physicochemical properties of the CmbHLH TFs [32].

### 2.3. Multiple Sequence Alignment, Phylogenetic, and Conserved Motifs Analysis of CmbHLH Family Proteins 

To analyze the phylogenetic relationship of CmbHLH proteins, the conserved motifs of the CmbHLH proteins were searched and analyzed using the MEME online tool (http://meme.nbcr.net/meme/tools/meme, accessed on 15 March 2022) (search criteria: motif length was set to 10–100, the maximum number of retrieved motifs was set to 40, and the default parameters were used for the rest) [33].

Using the NJ method, a neighbor-joining phylogenetic tree was constructed based on the alignment of the identified CmbHLHs with AtbHLHs from *Arabidopsis* using MEGA 7.0 with 1000 bootstraps (the others with default parameters) [34]. Protein sequences of 156 AtbHLH family members were downloaded from TAIR (https://www.Arabidopsis.org/index.jsp, accessed on 12 November 2021) [35]. A phylogenetic tree was drawn using iTOL (http://itol.embl.de/, accessed on 9 November 2021) to group the CmbHLH proteins [36].

### 2.4. Excavation of Candidate CmbHLHs Resistant to Necrotrophic Fungi and Their Cis-Element Analysis

Based on the selection criteria for differential genes [37], the upregulated expression of *CmbHLHs* in response to *Alternaria* sp. innoculation were taken as potential candidate *CmbHLHs* resistant to necrotrophic fungi. Putative promoter sequences (2000 bp upstream of ATG) of the candidate genes were retrieved from the *Chrysanthemum* genome database (http://www.amwayabrc.com/zh-cn/index.html, accessed on 9 May 2021) (Table S1). *cis*-acting elements in the promoter sequences were predicted using the online software PlantCARE (http://bioinformatics.psb.ugent.be/webtools/plantcare/html/, accessed on 10 May 2021) [38]. The results were visualized and mapped using TBtools software v0.6673 [39]. 

### 2.5. Isolation and Arabidopsis Transformation of CmbHLH18

The total RNAs of ‘Huaiju 2^#^’ leaves were extracted using an RNAiso Plus kit (TaKaRa, Beijing, China) and purified using an ND-ONE-W spectrophotometer (Thermo, Waltham, MA, USA) according to the manufacturer’s protocol. Reverse transcription into cDNA was performed using a HiScript II 1st Strand cDNA Synthesis Kit (Vazyme, Nanjing, China) in a 20 μL reaction. cDNA was synthesized using specific primers (Table S6) with 500 ng of total RNA as the template.

The cDNA was used as a template and primer pair (CmbHLH18-F and CmbHLH18-R) (Table S6) were designed to amplify the coding area of *CmbHLH18*. The PCR program was conducted as follows: 5 min at 94 °C; 35 cycles of 94 °C for 30 s, 56 °C for 30 s and 72 °C for 60 s; 10 min at 72 °C. 

The coding DNA sequence of *CmbHLH18* (GenBank accession number: OP 313866) was constructed into the Super1300-35S vector [3]. The recombinant vector was introduced into *Agrobacterium* strains and transformed into *Arabidopsis* plants to produce plants overexpressing *CmbHLH18* using the floral dip method [40]. After three generations of Hygromycin B resistance screening, a small amount of *Arabidopsis* leaf genomic DNA was extracted for PCR amplification using the cetyltrimethylammonium bromide (CTAB) method, and a PCR reaction system (50 µL) was prepared consisting of 100 ng of template DNA, 2 µL of primer super1300-F, 2 µL of primer CmbHLH18-V-R, 25 µL of 2× Pfu MasterMix (Dye) (Vazyme Biotech, Nanjing, China), and 19 µL of ddH_2_O. The PCR reaction program consisted of the following steps: 94 °C for 5 min, 35 cycles of 94 °C for 30 s, 56 °C for 30 s, 72 °C for 60 s, and finally, 72 °C for 10 min. Electrophoresis using 1% agarose was performed to separate the PCR products, and a gel imaging system was used to detect and identify transgenic *Arabidopsis* plants. This was also identified by RT-qPCR with the primer pair of CmbHLH18-At-F and CmbHLH18-At-R (Table S6), following Section 2.9 in this part.

### 2.6. Inoculation of Necrotrophic Fungi and Sampling

According to the method of Zhao et al. [3], *Alternaria* sp. and *A. brassicicola* were activated. When plants of ‘Huaiju 2^#^’ grew to 8–10 leaves, the middle and upper leaves were punctured with a needle (approximately 0.41 mm diameter) and inoculated with spores of *Alternaria* sp. [3]. When *Arabidopsis* plants grew to 4 weeks, the spore solution of *A. brassicicola* was sprayed on leaves until droplet runoff occurred [41]. The two spores were suspended and diluted to a concentration of 10^7^ spores per milliliter in sterile distilled water (SDW) using the hemocytometer technique. After inoculation, plants were kept in a dark incubation chamber for 48 h at 25 °C with 100 % relative humidity (RH). Then, the plants were grown in the greenhouse with 40–60% RH, 20–25 °C, 60 mE·s^−1^ m^−2^ light intensity, and 14 h light/10 h dark light cycle for ‘Huaiju 2^#^’ [3], 10 h light/14 h dark light cycle for *Arabidopsis*.

Leaves of ‘Huaiju 2^#^’ were collected as samples at 0 d (before inoculation) and 1, 3, and 5 days post inoculation (dpi) with *Alternaria* sp. for expression profile analysis of candidate *CmbHLHs*. Leaves of the transgenic and WT *Arabidopsis* lines were collected at 0 d (before inoculation) and 1 dpi, 3 dpi, and 5 dpi with *A. brassicicola* for gene expression analysis and activity determination of antioxidant enzymes and defense enzymes. 

### 2.7. Morphology and Disease Severity Index (DSI) after Inoculation with A. brassicicola in CmbHLH18 Transgenic Arabidopsis Lines

We divided the infected leaves into five grades following the method of Xu [42]: grade 0 (lesion size ≤ 5%), grade 1 (5% < lesion size ≤ 25%), grade 2 (25% < lesion size ≤ 50%), grade 3 (50% < lesion size ≤ 75%) and grade 4 (lesion size > 75%). At 5 dpi, 15 plants from each line were randomly selected, and the number of leaves of each grade was counted. The DSI was calculated with the following formula.

$$\mathrm{DSI}=\frac{\sum \left(\mathrm{Grade}\;\mathrm{of}\;\mathrm{disease}\;\times \;\mathrm{Number}\;\mathrm{of}\;\mathrm{diseased}\;\mathrm{leaves}\;\mathrm{of}\;\mathrm{this}\;\mathrm{grade}\right)}{150}\times 100\%$$

### 2.8. Histochemical Staining and Microscopic Analysis after Inoculation with A. brassicicola in Arabidopsis and CmbHLH18 Transgenic Arabidopsis Lines

Evans Blue [43] was used to observe necrotic cells at 0, 5 dpi and fungal growth at 3, 5 dpi. Aniline blue staining [44] was used to observe callose deposition at 0 and 0.5 dpi. At 0 and 0.5 dpi, nitro blue tetrazolium (NBT) and diaminobenzidine (DAB) staining were used to detect the superoxide anion (O_2_^−^) and hydrogen peroxide (H_2_O_2_) of the leaves, respectively [45].

### 2.9. Determination of Enzyme Activity and Gene Expression Analysis of Arabidopsis Lines

Following the literature, we quantified the activities of antioxidant enzymes, including superoxide dismutase (SOD) and catalase (CAT), and defense enzymes, including phenylalanine ammonia lyase (PAL), peroxidase (POD), chitinase (CHT) and *β*-1,3-glucanase (GLU) in leaves of transgenic and WT *Arabidopsis* plants were measured following Liu et al. [46].

The method of obtaining cDNA is the same as 2.5; cDNA was used as a sample for the RT-qPCR of genes. RT-qPCR was done using an AceQ RT-qPCR SYBR Green Master Mix (Vazyme, Nanjing, China). *CiUBI* (Cse2.0_LG8) was used as an internal reference for the expression level of *CmbHLH18* in various tissues of *Chrysanthemum*. *AtActin* (823805) was selected as an internal reference for the expression level of genes post-inoculation in *Arabidopsis*. Specific primers were designed using primer 5.0 software [3] (Table S6). The expression levels in RT-qPCR were calculated using the 2^−△△CT^ method [47].

### 2.10. Statistical Analysis

A one-way ANOVA and Student’s *t*-test were performed using SPSS 18.0 software (IBM, Armonk, NY, USA). One-way ANOVA tests were performed to test the DSI of the *Arabidopsis* lines after inoculation of *A. brassicicola*. Student’s *t*-tests were used to test the gene expression level analysis and activities of antioxidant and defense enzymes after *Alternaria* sp. inoculation.

## 3. Results

### 3.1. Identification of the CmbHLH Transcription Factor Family

#### 3.1.1. Hydrophilicity and High Aliphatic Amino Acid Content in 71 CmbHLH TFs Identified in *Chrysanthemum*

Our sequence analysis identified 71 proteins with a typical bHLH domain (Figure S1) and designated as CmbHLH1, 2, 3… 71, and their physical and chemical properties are summarized in Table 1. For these 71 CmbHLH proteins, the predicted polypeptide sequence lengths ranged from 158 to 937; the predicted molecular weights ranged from 17.67–103.49 kDa, and their theoretical isoelectric points (pIs) ranged between 4.75–9.2. Among them, the pIs of 15 proteins were greater than 7.5, while the pIs of 46 proteins were lower than 6.5. Most (64.8%) of these CmbHLH proteins were rich in negatively charged amino acids. The results of the protein hydropathicity analysis showed that the grand average of hydropathicity (GRAVY) of all 71 sequences was lower than 0, which indicated that the bHLH proteins in *Chrysanthemum* are generally hydrophilic proteins. The greater II (the instability index) value, the more unstable the protein. Therefore, most of the CmbHLH proteins were apparently unstable; the least stable of them was CmbHLH3 (CHR00034167-RA). Furthermore, these CmbHLHs had relatively high aliphatic amino acid (AI) contents, with CmbHLH22 (CHR00078227-RA) containing the highest number of AIs. Taken together, these analyses showed that *Chrysanthemum* harbors a wide array of bHLH TFs with hydrophilicity and high aliphatic amino acid content.

#### 3.1.2. Phylogenetic Differences between Groups of bHLHs from *Chrysanthemum* and *Arabidopsis*

**Figure 1:**
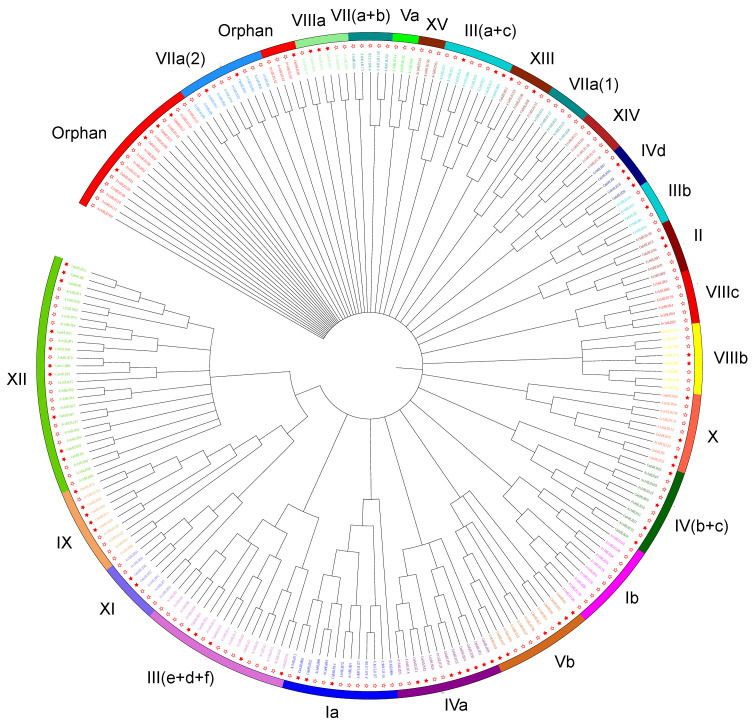
Phylogenetic relationship of the bHLH TF family among *Arabidopsis* and *Chrysanthemum*. Different colored arcs are used to distinguish different groups or subgroups. The hollow star and red solid star represent *Arabidopsis* and *Chrysanthemum*, respectively.

Phylogenetic analysis of the DNA-binding domain of the 71 CmbHLHs as well as 156 bHLHs from *Arabidopsis* suggested that there were 17 groups, none of which belonged to clade Ib, Va, VIIa (1), VII(a+b), VIIIc, XIV, and XV in *Chrysanthemum* (Figure 1, Table S2). Five bHLHs were unique to *Chrysanthemum*, resulting in their classification as “Orphans”. These results showed that the CmbHLH TF family probably expanded and contracted during the evolutionary process in *Chrysanthemum*. Motif analysis of the CmbHLH proteins showed that 40 putative motifs among the 71 CmbHLH proteins were identified (Figure S2). Members of the same subfamily of CmbHLH had similar conservative motifs and numbers, and their location distribution was relatively conservative. This may help to analyze the phylogenetic relationship between bHLH TFs. 

#### 3.1.3. Domains of CmbHLHs Identified by Variation-Conserved Residues

**Figure 2:**
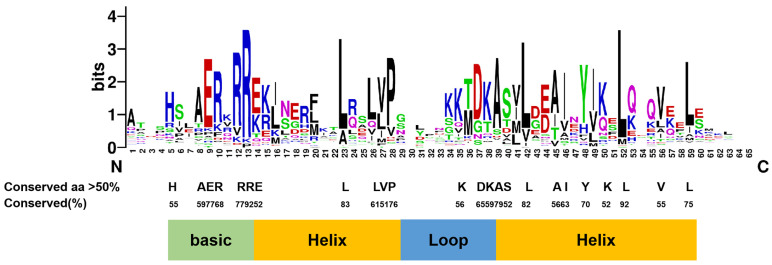
Sequence logos of the CmbHLH domains. The height of the amino acids at each site indicates the conservatism of the corresponding amino acids. Capitalized letters below the sequence logos indicate that more than 50% of amino acids are consistent in the conserved domain of all CmbHLH proteins.

To better understand the function of the 71 CmbHLH TFs identified in *Chrysanthemum*, their domains and DNA-binding capacities were analyzed. There were 24 amino acid residues that were highly conserved in >50% of the candidate proteins (Figure 2), and the R13, L23, L42, and L52 residues were conserved in >80% of the proteins (Table S3). The results of the domain analysis suggested that these conserved sites were likely involved in bHLH function. Further analysis showed that the basic regions of CmbHLHs were composed of 10–13 amino acids and contained a typical H5-E9-R13 sequence motif (His5-Glu9-Arg13) for binding DNA, which is necessary for bHLH to function as a transcription factor [48]. In particular, E9 and R13 residues have a function in the recognition and binding of E-box motifs in the promoter regions of target genes.

### 3.2. Excavation of Candidate CmbHLHs’ Resistance to Necrotrophic Fungus Alternaria

Five CmbHLHs were highly upregulated in the transcriptome (Accession number: PRJNA448499) when plants were inoculated with *Alternaria* sp. and were potentially involved in the resistance to necrotrophic fungi. The five candidate genes were named *CmbHLH16*, *CmbHLH18*, *CmbHLH28*, *CmbHLH30*, and *CmbHLH60* (Table S4). CmbHLH16, CmbHLH28, and CmbHLH30 belong to the IVd subfamily, CmbHLH18 belongs to the IVa subfamily, and CmbHLH60 belongs to the IX subfamily. They contain a total of 15 motifs (Figure S2).

#### 3.2.1. Analysis of Promoters of the Five Candidate *CmbHLHs* 

To comprehend the transcriptional regulating function of the 71 CmbHLH TFs identified in *Chrysanthemum*, taken the five UP *CmbHLHs* as representative, sequence analysis identified 2000 bp fragments upstream of these five genes with their respective sequences in the *Chrysanthemum* genome database (http://www.amwayabrc.com/index.html, accessed on 9 May 2021). Then, a promoter analysis was conducted to identify the potential function of *cis*-acting elements within these regions (Figure S3; Table 2) and found that three major types of *cis*-elements were present in the promoters of the UP *CmbHLHs*. The first type is responsive to plant hormones and includes ABRE, AuxRR, CGTCA/TGACG, P-box, and TCA elements, which are sensitive to ABA, IAA, JA, GA, and SA, respectively. The second type is responsive to defense and stress, including TC-rich repeats (defense and stress responsiveness, which are only in the promoter of *CmbHLH18*), MBS (drought stress), LTR (low-temperature stress), and ARE (anaerobic stress) elements. The third type participates in plant growth and development, such as that of the GCN4_Motif in endosperm expression. Our finding of hormone-responsive and defense-responsive promoter elements suggested that the *CmbHLHs* may have been upregulated specifically through these elements during *Alternaria* sp. infection and prompted us to investigate whether these elements could be related to an enhanced disease resistance phenotype.

#### 3.2.2. Expression Analysis of the Five up *CmbHLHs* in Response to Necrotrophic Fungus *Alternaria*

**Figure 3:**
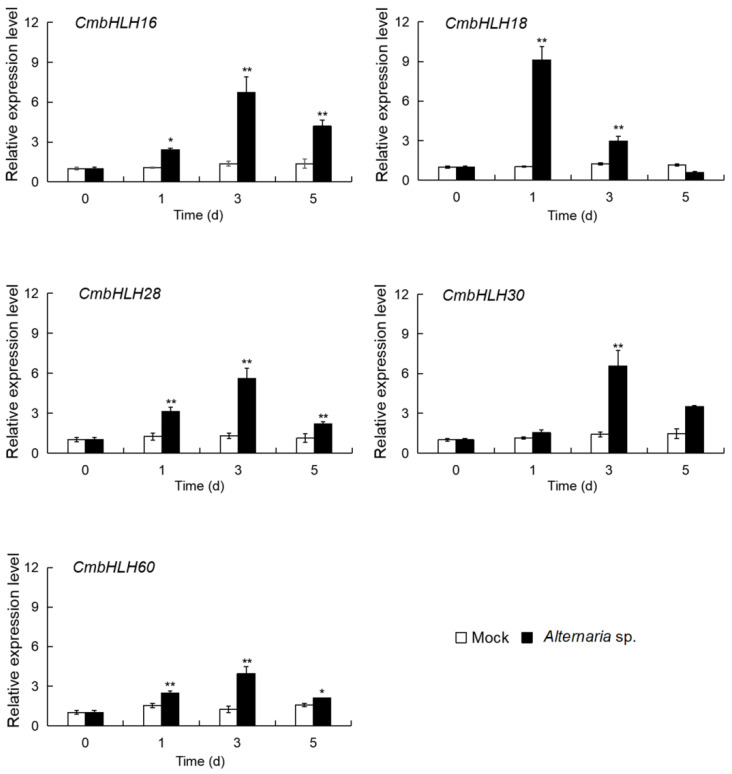
Expression level analysis of the five UP *CmbHLHs* after *Alternaria* sp. inoculation. Asterisks represent statistically significant differences for each time interval between *Alternaria* sp. versus Mock using the *t*-test (*n* = 3; *, *p* < 0.05; **, *p* < 0.01).

In order to better understand whether and how these promoter elements may contribute to transcriptional regulation of these five UP *CmbHLHs* to mediate a disease-resistant phenotype, we next inoculated *Chrysanthemum* with *Alternaria* sp. Then, their expression levels were examined (Figure 3). The results showed that these *CmbHLHs* were significantly induced by inoculation with *Alternaria* sp. compared with Mock. More specifically, *CmbHLH18* expression peaked at 1 dpi and was 9.12 times higher than that of the Mock, whereas the expression of *CmbHLH16*, *CmbHLH30*, *CmbHLH28*, and *CmbHLH60* reached their highest levels at 3 dpi (reaching 6.72-, 6.58-, 5.61-, and 3.93-fold, respectively, that of the Mock).

These results indicated that these five UP *CmbHLHs* might play a positive regulatory role in response to *Alternaria* sp. In particular, the expression of *CmbHLH18* was strongly upregulated by *Alternaria* sp. (the necrotrophic fungus) infection. Therefore, *CmbHLH18* is mostly expected to become a candidate gene resistance against the necrotrophic fungus *Alternaria* in plants.

#### 3.2.3. *CmbHLH18* Enhances Resistance against Necrotrophic Fungus *Alternaria* in *Arabidopsis*

**Figure 4:**
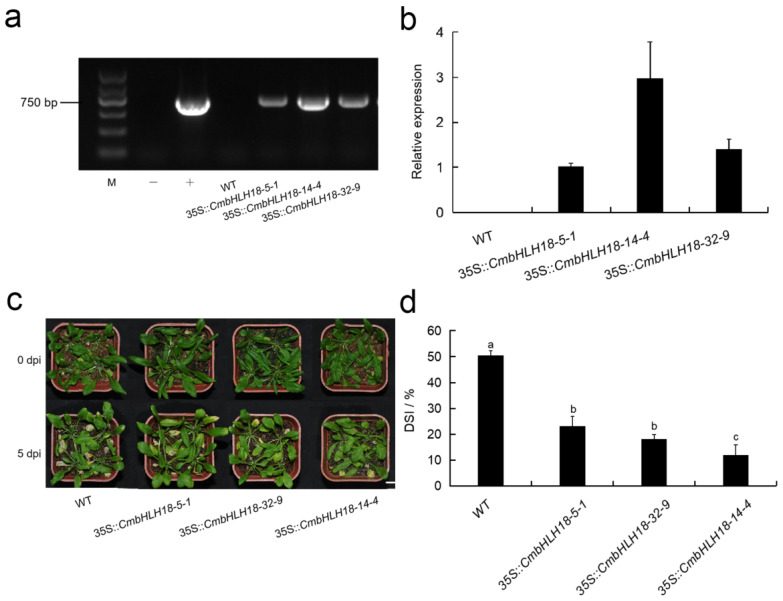
Identification of *CmbHLH18*-transformed *Arabidopsis* plants and morphology and DSI statistics. (**a**,**b**) PCR and RT-qPCR detection of *CmbHLH18* in transformed *Arabidopsis*. M: DNA DL2000 Marker; (**c**) Morphologic change of *CmbHLH18* transformed *Arabidopsis* lines after *A. brassicicola* inoculation (Bar = 1 cm); (**d**) DSI of different *Arabidopsis* lines at 5 dpi. The different letters signified significant differences at *p* < 0.05 by one-way ANOVA.

**Figure 5:**
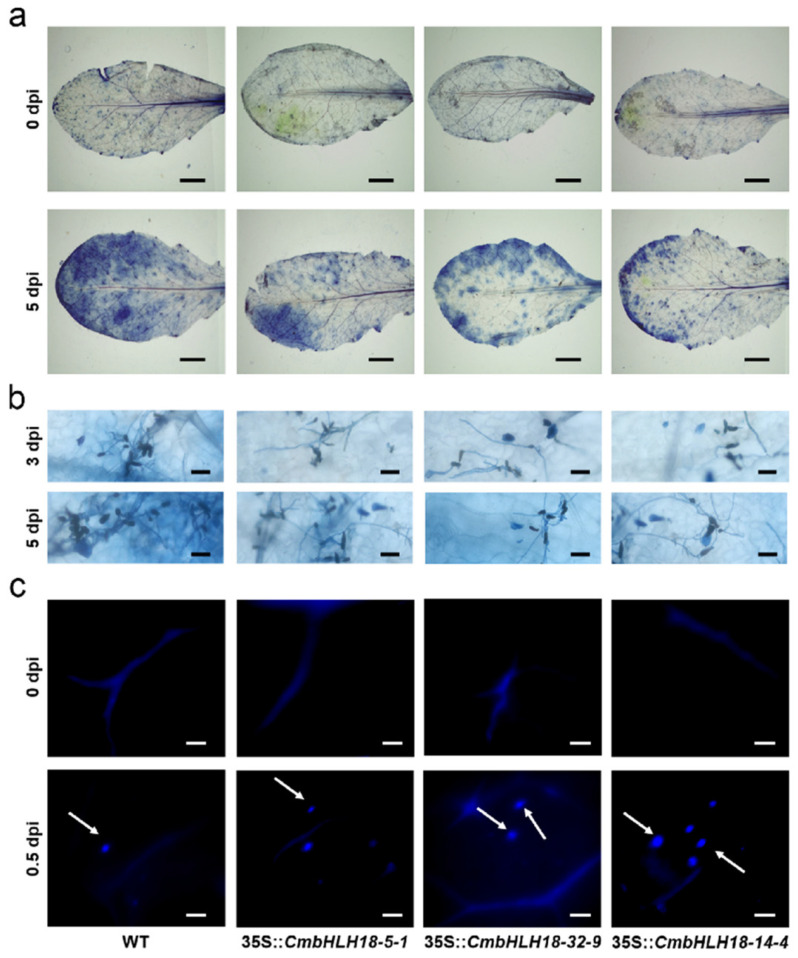
Effect of *CmbHLH18* on the cell activity of leaves, growth of fungi, and callose deposition of leaves in *Arabidopsis* after inoculation with *A. brassicicola*. (**a**) Microscopic visualization of necrotic cells in leaves (Bar = 1 cm); (**b**) observation of fungi growth (Bar = 50 μm); (**c**) Detection of callose deposition in leaves (Bar = 100 μm).

In order to further verify the function of *CmbHLH18* resistance against the necrotrophic fungus in plants, we isolated *CmbHLH18* (GenBank accession number: OP 313866) and transformed it into *Arabidopsis* (Col). Transgenic plants were identified using PCR and RT-qPCR (Figure 4a,b). Transgenic plants show PCR products of 516 bp, while normal plants contain the amplified region with 0 bp in size (Figure 4a). There were no significant differences in morphology between the WT and transgenic plants before *A. brassicicola* inoculation. However, at 5 dpi, DSI was significantly higher in the WT than in transgenic plants (Figure 4c,d). This is reflected in cell activities being higher in transgenic plants than the WT (Figure 5a, Table S5). 

The speed of spore growth of inoculated leaves at 3 dpi and 5 dpi was significantly slower in transgenic plants than in WT plants (Figure 5b). The leaves of transgenic plants showed more callose deposition compared with WT plants at 0.5 dpi with *A. brassicicola* (Figure 5c).

**Figure 6:**
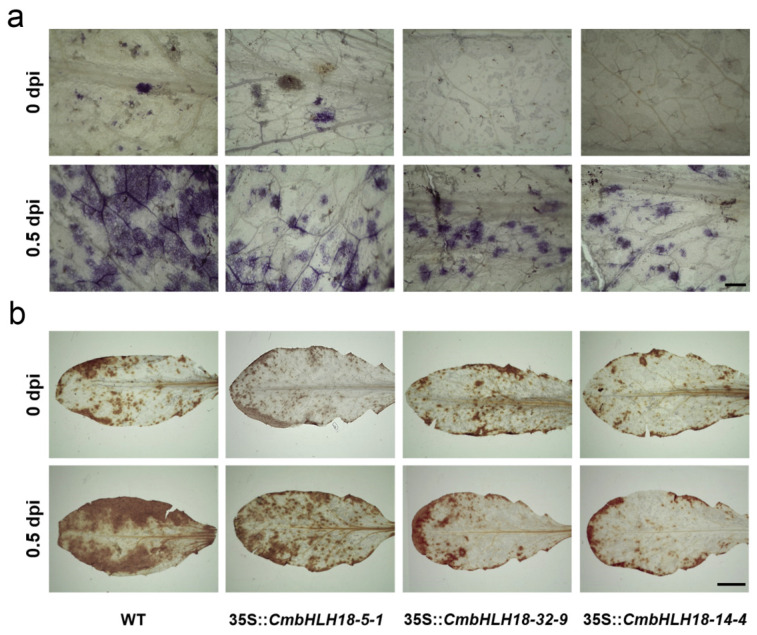
Effect of *CmbHLH18* on ROS accumulation in leaves in *Arabidopsis* after inoculation with *A. brassicicola*. (**a**) Accumulation of O_2_^−^ (NBT staining) (Bar = 1cm); (**b**) Accumulation of H_2_O_2_ (DAB staining) (Bar = 100 μm).

To further analyze the effect of *CmbHLH18* on ROS accumulation in transformed *Arabidopsis* leaves after *A. brassicicola* inoculation, NBT staining and DAB staining were conducted. Transgenic plants of *Arabidopsis* after *A. brassicicola* inoculation exhibited a lower accumulation of ROS than the wild type plants at 0.5 dpi (Figure 6), as indicated by the NBT staining and DAB staining.

**Figure 7:**
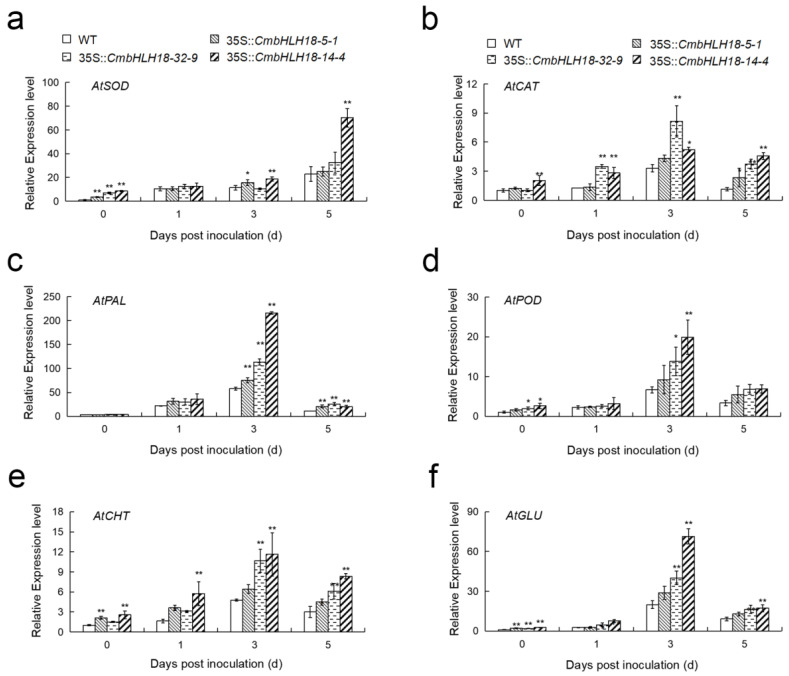
Effect of *CmbHLH18* on the expression level of antioxidant and defense enzyme genes in *Arabidopsis* after inoculation with *A. brassicicola*. (**a**,**b**) Expression levels of antioxidant enzyme genes; (**c**–**f**) Expression levels of defense enzyme genes. Asterisks represent statistically significant differences between different lines by *t*-test (*n* = 3; *, *p* < 0.05; **, *p* < 0.01).

**Figure 8:**
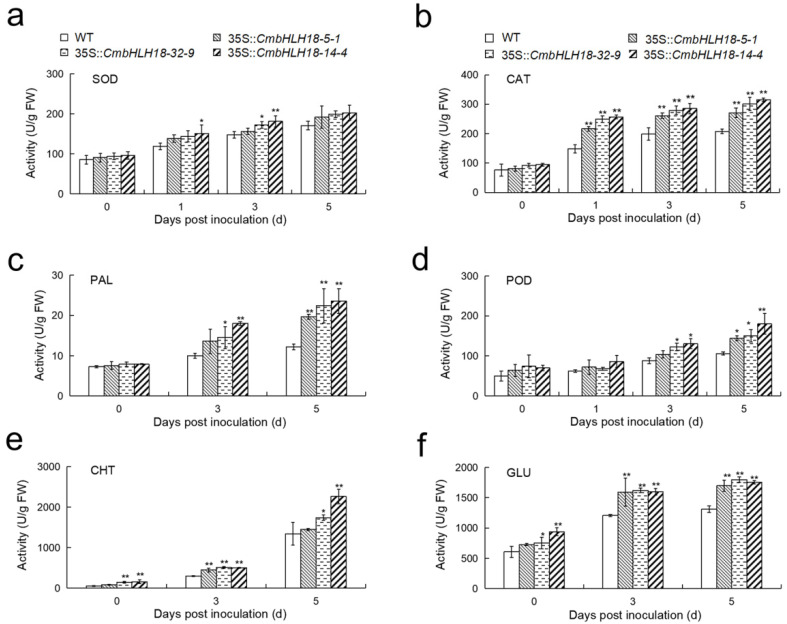
Effect of *CmbHLH18* on the activities of antioxidant and defense enzymes in *Arabidopsis* after inoculation with *A. brassicicola*. (**a**,**b**) Activities of antioxidant enzymes; (**c**–**f**) Activities of defense enzymes. Asterisks represent statistically significant differences between different lines by *t*-test (*n* = 3; *, *p* < 0.05; **, *p* < 0.01).

The profiling of gene expression and activities of enzymes involved in ROS scavenging and plant defense suggested that after inoculation, there was a significant increase in ROS scavenging and defense abilities in transgenic plants (Figure 7 and Figure 8). This indicated that *CmbHLH18* enhanced resistance to *A. brassicicola* by increasing the ability of ROS scavenging and the activity of defense enzymes in *Arabidopsis*.

## 4. Discussion

The bHLH TF family exists widely in plants and animals and is one of the largest transcription factor families in plants [26,27]. This study reports the gene family for the first findings in *Chrysanthemum*. Our sequence analysis of genes identified 71 bHLHs of 17 subfamilies in *Chrysanthemum*, which is significantly smaller than that reported in *Arabidopsis* (162 bHLH TFs into 21 subfamilies, [7]), white poplar (202 bHLH TFs into 25 subfamilies, [17]), and rice (167 bHLH TFs into 22 subfamilies, [49]). Therefore, gene duplications occur more in some species than in others, such as *Chrysanthemum*, sacred lotus [50], and watermelon [51].

In general, genes with the same or similar structures are clustered in the same subfamily and may have a similar function [52]. For example, TT8 of the bHLH IIIf subfamily in *Arabidopsis* and TaPpb1 of the bHLH IIIf subfamilies in *Triticum aestivum L.* can both regulate anthocyanin synthesis [19,53]. AtbHLH3 of the bHLH IIId subfamily in *Arabidopsis* and GmbHLH3 of the bHLH IIId subfamily in *Glycine max (Linn.) Merr.* can both regulate jasmonate-induced leaf senescence [54,55]. Therefore, cluster and comparative analysis of the phylogenetic tree classification results between CmbHLH proteins and the bHLH of other species with known functions can be helpful to predict the functions of some genes in *Chrysanthemum* (Figure 1). In other cases, there are some differences in function, even if these genes are clustered into the same subfamily. For example, NFL of the bHLH IIIb subfamily in *Arabidopsis* can promote flowering, but OsbHLH61 of the bHLH IIIb subfamily in *Oryza sativa L.* can increase resistance to the brown planthopper [56,57]. The GL3 of the bHLH IIIf subfamily in *Arabidopsis* can promote root epidermal development, which differs from the function of TaPpb1 in the same subfamily [19,58]. AtbHLH18 (belonging to Ⅳa) achieved iron homeostasis by promoting JA-induced degradation of the Ferritin (FIT) protein and inhibiting iron absorption in *Arabidopsis* [59]. There may be a great difference in function between CmbHLH18 and AtbHLH18 in function.

The Weblogo diagram obtained by multi-sequence alignment of the CmbHLH domain shows that the bHLH domain is conserved in all CmbHLHs, which is similar to the *Arabidopsis* bHLH domain (Figure 2). In the CmbHLH domain, two amino acid residues, Leu-23 and Leu-52, are relatively conservative, accounting for 83% and 92%, respectively. The two residues are necessary for the formation of a homologous or heterodimer [9]. This indicates that, on the one hand, CmbHLH proteins probably form homodimers by itself, and on the other hand, CmbHLH proteins probably also interact with other TFs, such as R2R3-MYB, to form heterodimers [9]. Understanding its domains will deepen our understanding of the function.

bHLH TF family has a variety of biological functions [26,27,28]. However, there are still many unknowns about the function of bHLH TFs in biotic stress. The comparison of the *cis*-acting elements on the promoters of the five *CmbHLHs* showed that there are *cis*-elements involving plant hormone responsiveness, stress responsiveness, and plant growth and development. SA plays a crucial role in plant defense and is generally involved in the activation of defense responses against biotrophic and hemi-biotrophic pathogens, as well as the establishment of systemic acquired resistance [60]. By contrast, JA is usually associated with a defense against necrotrophic pathogens and herbivorous insects [61]. Interestingly, the TC-rich repeats were unique to the promoter of *CmbHLH18*, which was located at 515 bp upstream of ATG and was involved in defense and stress responsiveness (Figure S3; Table 2). This may be related to the fact that *CmbHLH18* can significantly respond to the necrotrophic fungus *Alternaria* sp. infection in *Chrysanthemum*. Surprisingly, *CmbHLH18* may also enhance the resistance of *Arabidopsis* to *A. brassicicola* (Figure 4c), which is another typical necrotrophic fungus of *Alternaria*. 

Callose is generally believed to positively regulate the stress response of plants [62,63,64,65]. Under abiotic and biotic stress, plants often trigger the accumulation of callose [64]. Callose deposition in the cell wall, plasmodesmata, and sieve pores controls the cell wall permeability, prevents further penetration of the pathogen into the tissue, and reduces the loss of cellular water and solute to maintain the internal stabilities of cells [66]. In this study, leaves of *CmbHLH18*-transformed *Arabidopsis* had more callose than WT after *A. brassicicola* inoculation, indicating that *CmbHLH18* could enhance the defense of *Arabidopsis* against *A. brassicicola* by inducing callose deposition.

When plants are subjected to stress (including biotic and abiotic stress), a large amount of ROS (O_2_^−^, H_2_O_2_, etc.) is generated in the cells. If ROS is not cleared in time, it damages plant tissues. *CmbHLH18*-transformed *Arabidopsis* plants reduce the content of O_2_^−^ and H_2_O_2_ in response to fungal attacks. This is consistent with Yao et al. [67], who showed that overexpression of *FtbHLH2* reduced the amount of ROS and increased the endurance of cold stress in *Arabidopsis*. In this study, the heterologous overexpression of *CmbHLH18* enhanced resistance to *A. brassicicola* in *Arabidopsis* by decreasing the content of O_2_^−^ and H_2_O_2_ (Figure 6). 

Plants can scavenge ROS and be protected from oxidative stress damage through an antioxidant protecting system involving SOD and CAT [68]. Heterologous overexpression of *VvbHLH1* in *Arabidopsis* [69] and *NtbHLH123* in tobacco [16] both improved the antioxidant enzyme system and increased the antioxidant capacity of transgenic plants. Our results showed a similar pattern, suggesting that *CmbHLH18* could decrease the amount of ROS, reduce plant damage, and enhance resistance to *A. brassicicola* by activating the expression of antioxidant genes and increasing the activities of antioxidant enzymes.

Plants can resist pathogen invasion through lignification. PAL and POD are key enzymes in the lignin synthesis pathway [70]. CHT and GLU are pathogenesis-related (PR) enzymes that can destroy fungal cell walls and prevent the invasion of pathogenic fungus [71]. Studies have shown that the expression levels of PAL, POD, CHT, and GLU are significantly increased by *Magnaporthe grisea* in rice [72]. In this study, after inoculation with *A. brassicicola*, the activities of PAL, POD, CHT, and GLU in transgenic leaves were significantly higher than those in WT leaves (Figure 8), and the corresponding gene expression levels in transgenic leaves were also higher than those in WT leaves (Figure 7).

## 5. Conclusions

We first identified 71 CmbHLHs genes of 17 subfamilies, and the genes in ‘Huaiju 2^#^’ are generally hydrophilic proteins and high aliphatic amino acid content. Additionally, combining the results of RT-qPCR analysis after *Alternaria* sp. inoculation, we found that *CmbHLH16*, *18*, *28*, *30*, and *60* may be involved in resistance to biotic necrotrophic fungus. They all have hormone-responsive elements that are associated with enhanced disease resistance in their *cis*-acting elements, and *CmbHLH18* may be a gene resistant to necrotrophic fungus in *Chrysanthemum*. 

Heterologous overexpression of *CmbHLH18* can enhance the resistance to necrotrophic fungus in *Arabidopsis* by a series of physiological and genetic activities, inducing callose deposition and increasing the activities and gene expression levels of antioxidant and defense enzymes.

The identification of CmbHLHs in this study will help to further identify candidate genes for resistance to necrotrophic fungus, elucidate the molecular mechanism of resistance to necrotrophic fungus in *Chrysanthemum*, and use *CmbHLHs* to breed a new variety of *Chrysanthemum* with high resistance to necrotrophic fungus.

## Figures and Tables

**Table 1:** Physiochemical properties of the CmbHLH TF family.

Name	ID	aa	MW (×10^4^)	pI	II	AI	GRAVY
CmbHLH1	CHR00023546-RA	272	3.062	7.79	49.89	81.36	−0.436
CmbHLH2	CHR00043772-RA	307	3.483	6.41	52.99	73.65	−0.578
CmbHLH3	CHR00034167-RA	328	3.700	4.8	62.02	74.85	−0.588
CmbHLH4	CHR00082406-RA	244	2.729	5.99	28.9	80.7	−0.441
CmbHLH5	CHR00073020-RA	417	4.630	5.59	59.66	54.68	−0.792
CmbHLH6	CHR00087928-RA	411	4.581	6.82	57.63	61.39	−0.763
CmbHLH7	CHR00079719-RA	468	5.246	6.35	45.31	75.79	−0.551
CmbHLH8	CHR00010518-RA	480	5.293	6.09	47.99	58.06	-0.739
CmbHLH9	CHR00002838-RA	398	4.480	8.64	60.66	76.68	−0.589
CmbHLH10	CHR00029628-RA	609	6.830	4.92	47.81	87.87	−0.329
CmbHLH11	CHR00053551-RA	361	4.081	6.08	61.37	54.88	−0.923
CmbHLH12	CHR00027604-RA	278	3.093	8.41	29.44	80.22	−0.53
CmbHLH13	CHR00020071-RA	410	4.558	9.29	47.53	76.56	−0.437
CmbHLH14	CHR00001268-RA	306	3.452	5.73	61.53	78.73	−0.423
CmbHLH15	CHR00066657-RA	453	5.049	6.33	32.95	63.07	−0.728
CmbHLH16	CHR00085490-RA	937	10.349	5.36	51.72	70.52	−0.551
CmbHLH17	CHR00014650-RA	350	3.838	6.19	50.86	61.57	−0.693
CmbHLH18	CHR00061827-RA	280	3.096	5.07	52.56	74.18	−0.44
CmbHLH19	CHR00004349-RA	427	4.836	5.51	51.06	77.07	−0.594
CmbHLH20	CHR00030629-RA	291	3.261	9.08	60.77	56.98	−0.832
CmbHLH21	CHR00050993-RA	310	3.481	6.76	46.73	84.00	−0.414
CmbHLH22	CHR00078227-RA	201	2.288	6.3	48.15	109.6	−0.098
CmbHLH23	CHR00016706-RA	534	6.031	5.9	38.2	80.24	−0.398
CmbHLH24	CHR00089135-RA	376	4.183	4.87	52.25	66.68	−0.716
CmbHLH25	CHR00058509-RA	262	2.931	6.62	46.86	59.12	−0.844
CmbHLH26	CHR00075848-RA	169	1.871	8.3	54.21	76.21	−0.576
CmbHLH27	CHR00021504-RA	294	3.272	7.01	48.12	66.33	−0.805
CmbHLH28	CHR00085488-RA	567	6.347	5.99	59.9	85.31	−0.386
CmbHLH29	CHR00066393-RA	592	6.578	6.42	41.98	79.34	−0.503
CmbHLH30	CHR00049557-RA	191	2.134	6.2	45.33	90.79	−0.458
CmbHLH31	CHR00020620-RA	501	5.364	5.56	58.67	57.84	−0.734
CmbHLH32	CHR00040132-RA	399	4.398	5.3	55.48	59.65	−0.624
CmbHLH33	CHR00025933-RA	251	2.872	8.31	43.43	67.17	−0.74
CmbHLH34	CHR00067596-RA	319	3.529	6.32	39.39	87.12	−0.37
CmbHLH35	CHR00085567-RA	249	2.785	7.65	37.57	90.44	−0.363
CmbHLH36	CHR00032751-RA	326	3.705	6.47	51.81	80.77	−0.557
CmbHLH37	CHR00051320-RA	200	2.273	6	58.68	83.3	−0.385
CmbHLH38	CHR00052362-RA	277	3.206	6.51	48.97	86.86	−0.588
CmbHLH39	CHR00032387-RA	309	3.414	6.26	62	54.56	−0.984
CmbHLH40	CHR00068388-RA	211	2.379	4.75	55.92	42.99	−1.068
CmbHLH41	CHR00062368-RA	280	3.096	5.07	52.56	74.18	−0.44
CmbHLH42	CHR00005466-RA	319	3.574	6.77	46.83	78.84	−0.399
CmbHLH43	CHR00028154-RA	215	2.422	6.46	49.22	86.56	−0.66
CmbHLH44	CHR00041708-RA	322	3.517	5.84	44.78	58.73	−0.793
CmbHLH45	CHR00076371-RA	310	3.501	7.29	43.91	70.81	−0.701
CmbHLH46	CHR00027603-RA	368	4.127	6.07	29.5	78.59	−0.551
CmbHLH47	CHR00053214-RA	318	3.554	6.98	55.24	84.37	−0.614
CmbHLH48	CHR00031690-RA	572	6.428	5.22	43.38	83.50	−0.306
CmbHLH49	CHR00022486-RA	354	3.957	5.81	61.58	79.35	−0.638
CmbHLH50	CHR00077133-RA	398	4.381	6.1	58.84	71.28	−0.572
CmbHLH51	CHR00002812-RA	323	3.562	6.33	49.77	67.28	−0.541
CmbHLH52	CHR00091912-RA	360	3.774	5.98	48.34	64.28	−0.498
CmbHLH53	CHR00040106-RA	445	4.818	5.44	61.72	56.36	−0.612
CmbHLH54	CHR00053312-RA	240	2.746	7.01	35.36	71.88	−0.659
CmbHLH55	CHR00073646-RA	293	3.244	6.26	35.57	65.87	−0.744
CmbHLH56	CHR00078290-RA	391	4.097	5.98	50.74	69.44	−0.423
CmbHLH57	CHR00063973-RA	238	2.673	8.49	51.21	92.69	−0.329
CmbHLH58	CHR00023344-RA	255	2.752	5.28	56.8	69.65	−0.636
CmbHLH59	CHR00015822-RA	172	1.906	4.89	44.96	80.41	−0.412
CmbHLH60	CHR00050362-RA	517	5.822	9.16	49.15	56.44	−0.992
CmbHLH61	CHR00025279-RA	268	2.892	5.77	60.24	54.55	−0.83
CmbHLH62	CHR00061024-RA	418	4.680	5.69	51.93	85.50	−0.364
CmbHLH63	CHR00086566-RA	158	1.767	5.86	55.74	78.92	−0.717
CmbHLH64	CHR00076271-RA	240	2.699	9.2	33.72	85.21	−0.556
CmbHLH65	CHR00065282-RA	185	2.105	8.52	37.98	90.11	−0.437
CmbHLH66	CHR00005538-RA	317	3.586	8.59	59.45	85.52	−0.466
CmbHLH67	CHR00079802-RA	392	4.363	7.15	57.19	62.45	−0.627
CmbHLH68	CHR00060897-RA	244	2.806	5.07	61.31	85.08	−0.498
CmbHLH69	CHR00064856-RA	229	2.593	5.31	57.55	70.83	−0.717
CmbHLH70	CHR00031761-RA	327	3.679	8.15	51.81	64.71	−0.779
CmbHLH71	CHR00029899-RA	311	3.510	8.28	61.74	77.78	−0.446

Note: ID were obtained from *Chrysanthemum* genome database (http://www.amwayabrc.com/index.html, accessed on 9 May 2021). aa, protein length, MW (molecular weight; kDa), PI (theoretical isoelectric point), II (the instability index), AI (aliphatic index), and GRAVY (grand average of hydropathicity) were obtained from ExPASY (http://web.expasy.org/protparam/, accessed on 15 November 2021).

**Table 2:** Classification of *cis*-elements in the promoter sequences of the five UP *CmbHLHs*.

Items	Name of *cis*-Element	Sequence	Position from Translation Start Site	Function
Hormone response	ABRE	ACGTG/CACGTG /GACACGTGGC	+477	*Cis*-acting element involved in the ABA responsiveness
	CGTCA-motif	CGTCA	−1760	*Cis*-acting element involved in the MeJA-responsiveness
	P-box	CCTTTTG	+1172	GA-responsive element
	TCA-element	CCATCTTTTT	−905	*Cis*-acting element involved in SA responsiveness
	TGACG-motif	TGACG	+1760	*Cis*-acting element involved in the MeJA-responsiveness
	AuxRR-core	TGTCTCAATAAG	−287	*Cis*-acting element involved in auxin responsiveness
Defense and stress response	TC-rich repeats	GTTTTCTTAC	−515	*Cis*-acting element involved in defense and stress responsiveness
	ARE	AAACCA	−1728 − 1711 − 1162 +338	*Cis*-acting element essential for the anaerobic induction
	LTR	CCGAAA	+1616 + 1963	*Cis*-acting element involved in low-temperature responsiveness
	MBS	CAACTG	−1234	MYB binding site involved in drought-inducibility
Growth and development	GCN4-motif	TGAGTCA	+1694	*Cis*-regulatory element involved in endosperm expression

## Data Availability

The datasets supporting the results of this article are included within the article.

## Supplementary Materials

The following supporting information can be downloaded at: https://www.mdpi.com/article/10.3390/genes14020275/s1, Figure S1: Multiple sequence alignment of CmbHLH family proteins. The four-color modules at the top of the figure are represented, respectively: red represents the N-terminal basic region of bHLH, yellow, and green represent two amphiphilic regions, and blue represents a variable length loop. Process the data into pictures using Clustal W. Figure S2: Motif composition of the CmbHLH TF family. (a) Phylogenetic tree of CmbHLHs; (b) conserved motifs of the CmbHLHs; (c) sequence composition of each motif in CmbHLHs. Figure S3: Visualization of *cis*-elements in the promoter sequences of *CmbHLHs*. Different colors represent different *cis*-acting elements in the promoter sequences of *CmbHLHs*. Table S1: Promoter sequences of candidate resistant genes to necrotrophic fungus. Table S2: Number of bHLH TF in each subfamily of *Chrysanthemum* and *Arabidopsis.* Table S3: Proportion of conserved amino acids at each point of CmbHLH protein. Table S4: Selection criteria for differential genes. Table S5: Relative activity of leaf cells of WT and transgenic *Chrysanthemum*. Table S6: Primers used in this study.
