# Supplementary material for: Identification of CmbHLH Transcription Factor Family and Excavation of CmbHLHs Resistant to Necrotrophic Fungus Alternaria in Chrysanthemum
Source: Genes (Basel). 2023 Jan 20;14(2):275. doi: 10.3390/genes14020275 (PMC9957535; doi:10.3390/genes14020275)
Supplement: Supplementary file 1 [file genes-14-00275-s001.zip › genes-2146251-supplementary.pdf]

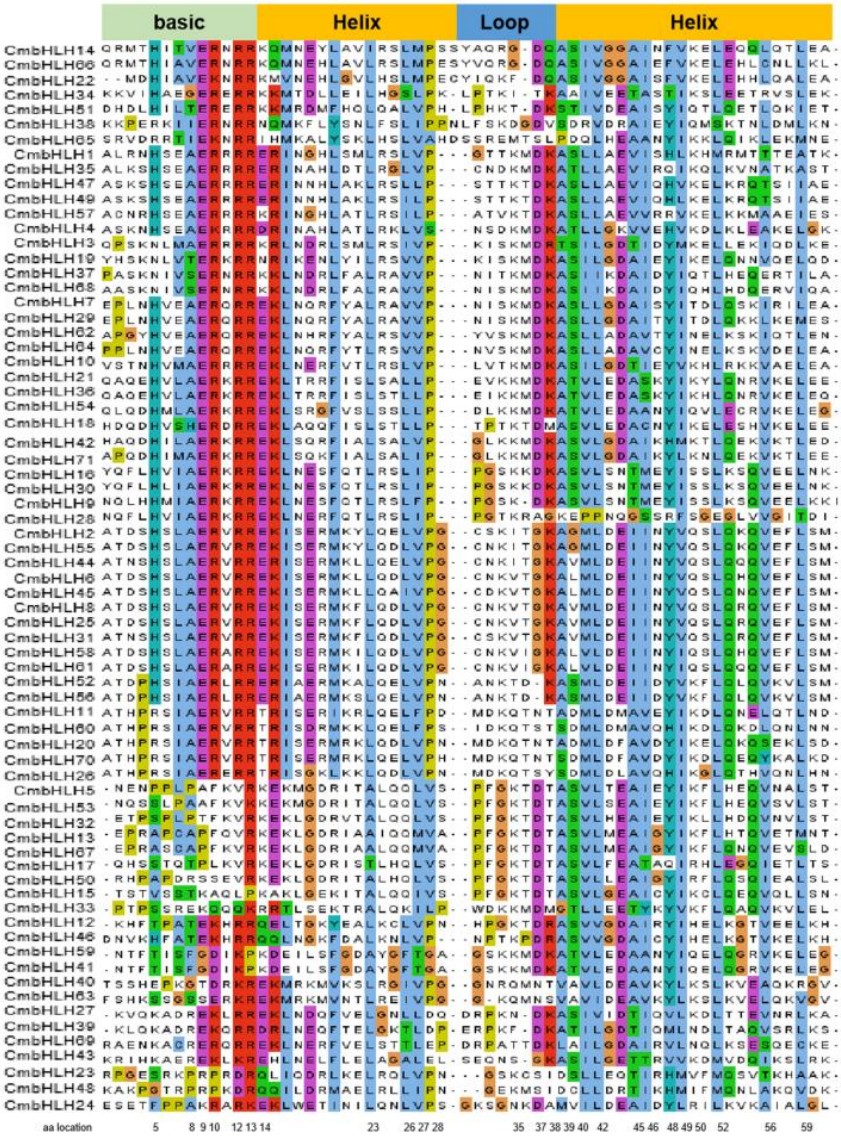

Figure S1 Multiple sequence alignment of CmbHLH family proteins. The four-color modules at the top of the figure are represented respectively: red represents the N-terminal basic region of bHLH, yellow and green represents two amphiphilic regions, blue represents variable length loop. Process the data into pictures by using Clustal W.

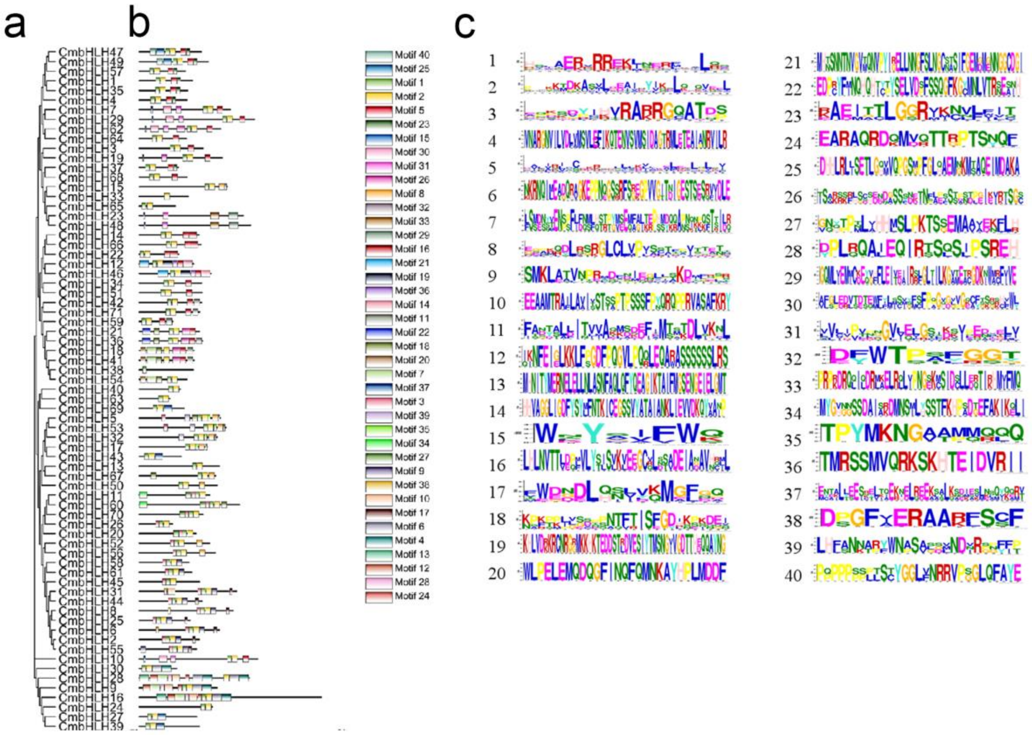

Figure S2 Motif composition of CmbHLH TF family. (**a**) Phylogenetic tree of CmbHLHs; (**b**) Conserved motifs of the CmbHLHs; (**c**) The sequence composition of each motif in CmbHLHs.

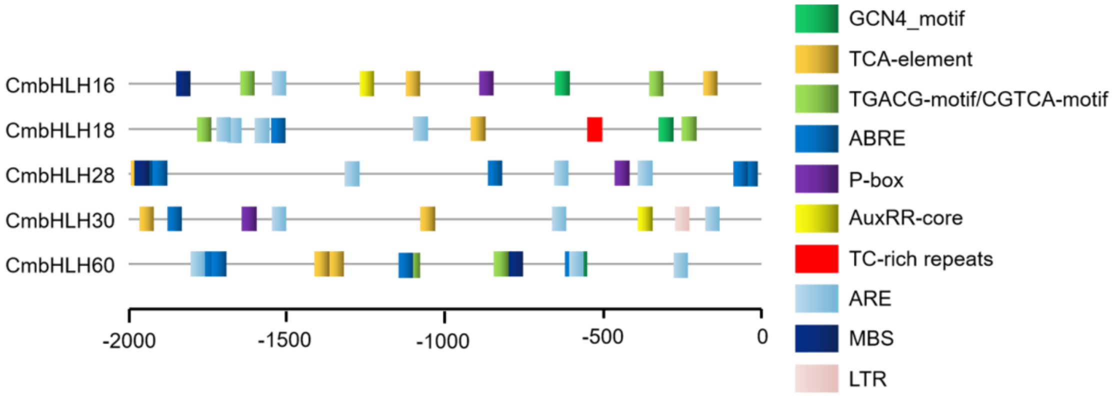

Figure S3 Visualization of *cis*-elements in the promoter sequences of *CmbHLH* genes. Different colors represent different *cis*-acting elements in the promoter sequences of *CmbHLH* genes.

Table S1 Promoter sequences of the candidate resistant genes to necrotrophic fungus.

**Promoter sequences of the candidate genes**
>*CmbHLH16*
TAGTCTTGGTTAGTAAACTTGGCTATTTAAATATGTTGTAAAAGTTGATTAAATATATACACAAG
TATATTGATCGCGCTATTATTTGTTCATCATTTTTTTATATCACTACTTGATATGATGGTTAAGTTG
CAGCATCAACTTGGACATATATTTATCACCACATCCTCAAGTGAATTTTCATGTTGTTTTGAGAT
CTTTCCTTAAGTACTAAATGTATTCTCTTTTTGCCACATATAAAATAATGGAGTAGAACTAAAAG
AAAAAGAAAATTAATTATATAAATATAACCTCCAACCGTATCAAAATTATTGTCGGGAGCGAA
GAATTAATAAAAGCGGGGCCAGTTATAGCTGCAGCTAAACAATATAAATATTCTCTATGGGTAT
ATTCTTTTTTTTTAACTTTTCGTAAAAAAGTGAAACAATTAATTGGATATCATAAAAGAAATATA

ACGATTATATGAGACGGATGGAGTAATATTTTATTATAAAATTGATTTTGTTATCATTAAAATG
ATTAAGATCGTGCAATAAATGTCAAGTTGTATGGTACGAGGTACATTTTTGTACTAGACAATCT
GAACATAATTGACAAAGAGGACCCAATGATGTGATAAAGAAGATTCAATGGTGGTGTTCTATT
GTTGACCTTCCTGACTCATTATCTCAAGACCATGATTGGGGACTAAATTAAATTGCGTTTTGCA
GCATATATGTATACAAACATATTCGTAAACATTTTTTTTTAAACATAGCCCTAAAAGTTGTGTT
CAAAGTATTCACACATGAAATAATTGTTTGTATCTTTACCGTTTACTCTCTATAAATTACCTTTAC
TCTCAGTAAATTACCTCTTTCAATCGACTTTATTTATGACCAGATGAACATCTTTTTACATTTTAA
ACTTAACCCTAAATGCTATATATCGGATTTTTTTTTACATGTGTAACAAGCATATAAACACGCGT
GTACTATTTGAAAGGGTTTTAACTATGTCTTTTTACCAGAAATAACTTATGTAAAAAGTCGTAC
AGAAAGCAATATATCCTACATAAAAGTGCAGAACTGATTATCTTTATGTGGATCCCATATCTTT
CAAATGGGCCTCTATACATGTCTTTTTTTATAACTTGCTTTATGATTAAAACCCTATTTGAAAAA
AAGTAGCCCTTTTGTTGAACAACGGCTATTATACATGTGGGCTAATTAGTCGCTATCAATTATCA
AAGGAGGAAATAACTTATAGAAAAGTATCAATATAATGGACCATCCATGGAAGAAGAAATCA
TCAAACAAATACCACGGTTGAATTTCAATCTATGACAAAATACTCCTTCTGATGTCATCTTTCTT
AATATATGCACATGTGTTTATGCATATATATACACCAACCCTAAAAATTTCAACAGGGTTTCTGT
AAACAAAACTTATTAAAAATGGACTCCATTTTCAATCTTGAAGAAACAAAAAGATCTCGCTTG
CTTCGACAAATCATGGACTCCTTTGGTTTCACTTACATTTGCTTATGGTCTCACTTTTCTCAGCC
TTCCAAGTACATCTCTCTCATTCAAGCTCAATTCTTTTTATATAGTATTCTTTGCAGTTCAAAAA
GAAATGTTTAATCATCTTTGATACCCATGCACAAATTGTAGTTGTTTGATCTTCATTGACGGAGT
ATACAAAGAAGGAAACAATCAAGCAAGCACATCATCTGGGAGTCTTTCCGTGAAGTCATTTC
TTGACTACAAAAAGTCGGTGTTCTTGATTGATAATTATAGTGGGTAAGAACAATAAAAACACA
ATCTTTTTTCGTATAATGAGTATTACTAAAACTCAGTTGTATGCGAGGGGTTTAACTGAATAAT
TAATGTTTTTTTATTAGAGGAGTTCCTGGGTTTGCATTCATGCACAATATTACATACATGGAGCG
GAATGAGTTGGAGCTCTTAAATCTGGCATCAAATTTTGCACAGCTACAATTTTATCAAGTAATC
ACACTTCAT
>*CmbHLH18*
GTGTAGTCTTTTGGAGTAAAAACCTAAACCGAACCAAGGCTGTCTTGTACAGTATTTTTAGGG
TTCCAAACCCTATCTTACCACAAGATAAGTGCTTTACTTCATTAATTCAATTATGTCTTCAATTAT
TTGTGTGTATGCTTGCATGTTCTCCATGTCCGGCTAATTTACTTGTGTGTTTGCTTAGGGAATATG
AAACTTGATTATTGAATATTGATTGGTTGTCTCTTATCTTAATTGTTAATGATCTAGTAATGCGAT
AGGTCATTTGTTGTGTAGTTAATGCTTGATTAATCTTTGTGAACCTTAGGCTATAACACCTAGAG
TAGTTGTAAGCTAAACCATCCAAAGAGTACCTTAGAGTTAGAGTTTATAATTCCTAGAGATTTT
GTAGACGAGACCCTTTGGTAGTTTCAAAGAGTTAGGGATAAACGCATACCCTACGACTTTAGA
CACACAACGCGATTAAGAATAAGGACGTGACAATTCAACCATCAATAGGAGATAATTAAGGA
GTAATAAAACCTAAATAAACACATCTTTAAACTATCATACGAGCACACAATAATTAGACATAG
ACTACATACACCTTAGACTAGATAATTAGACAAGTAGAGTAATCGGTAGAAATACCAACCCCC
AAATCCAAACCCCGAGATTCGTATCTTTCTAATTCCTAAAAAACATAAAAAGATAAAAAATAA
TCTTCCTCAACCCCATCCTTAGCGTAGTAACTTCTCACTAGCATTCCTATTCCCCTAGAGAGCG
ATATCAATACCCGTGGCTTACCCTAATACTATACTACAACGATTAGGTTCACTGCCTAGGGTGTT
ATAAAACTTGCTCGAAGAAAAGGAAGTTCCTTTGACCCCGAACGCGCTGCGTAAGCGTATCA
GGTATTCTTGAAAAAGAGGGTTATACTATGAAGATGCAAAGGGGTACAGTAAAGGTGATCAA
AGGTTGTCGGGTTATGATGATTGAGATCAGGAGAAAGAATTGTGTATATACTCTGGAAGCAAA
AGTGATGACATTTAGTGTACAAAAGCATGAAGATTCAAAACAAGTTGGATTCGAGAAACTTG
GTTCTAGACAAGTTAGGTTCAAACAACTTGGTCCCGGTGCTAAAACAGAAATCTATGGAATAC

ATGATGAAAAACATTTTTGGTTTATGGTGGAACGCGAGGAAGCTGGGGTTATTCAAGATAGTA
ATGATAATGTTGCAGTAACTCAAAGACAGTTGGAGGATAAGTAACTTGAAAGAAAAGAAAA
AAAGGACTGATTTGCAAAGGAGCATGAAAGGTTAAACCTTGGTGCAAAGTGGGGGCGCACA
CTATAAAAACTAGAGTACCCGGTTAAGAGGATGTAGTAGAGGGTAATGTTGTTGGGGAAAAA
AAAAAGAAATGGAAGGAGTCTAAGAAAGCCAATATAGGAAAATTACTGAAGTACAATGCCT
AGTCGACAAGGTGGTCCTCGCTTCAAGGTTCCAGTACAAGGATTAGTTATATTTAAGTGTTACT
CCAAAGATGAAAATTGTAGAACATTAGGAGCAAGCTTCACATAGATTAGGGGTTTAAGTGTAC
AATTAATTAGATTAGGTTTAGACTATAACTAGGATTAATTTGCTTGCCTTGTAATGCAAGTAAAA
AATATAATGTAAGCGTTTGAGGCGTTTGAGGTTACGCGTGTGGTTCTCTTGAGTCAATATGCGC
GCTGGTTTTCAAAGTTTGTTGGTTTTTTATCCAACAAAATTAAAATGGATATGACGCCAAAATG
AAAGACCTGTGAGTAGGTGTCCAAATCATCCCAGAAAATATAAATTTCAAAGTGGCTCATACA
TGCCTTTTTGTAAGGCTTCGGGTCAAGATTGCCAACTTTATTTTTTATTAAAAGATTCCTTTAAA
TTCTAGCAACCTTATAAGAACATAGCACCATTGTTTAACAAAATGTCTTCAATTTCTACACCCC
TAAAATTTTGAGGCTATACTGGTTCAACAACATCTTTTAGATATCTAAAGCTTCAAGACAAAAT
CCTCGAACTAAGAAACACAAATTCTGATTAT

>*CmbHLH28*

TTGATAATTATAGTGGGTAAGAACAATAAAAACACAATCTTTTTTTGTATAATGAGTATTACTAA
AAATCAACTGTATGCGAGGGGTTTTAACAGAATAATATTTATTATTAGAGGAATTCCAGGGTTT
GCATTCATGCACAATATTACATACATGGAGCGGAATGAGTTGGAGCTCTTAAATTTGGCATCAA
ATTTTGCACAGCTACAATTTTATCAAGTAATCACACTTCATATTTAAGTACGTATAAGTTTAGTT
TCAATTATGTAAGATTTGAAGCTAATGTGAATCCCTATTTTATCTTGCAGGAAGCAGGAATAAA
GGTATCCGATCTAGGCTGCATGGACACGGACACTGTCCAAAGACGGCGATAAGCAAAATCTA
AAAATATAAGACCTTACATGGACGGGAATTTATATATATATATATATATATAATTCAAAAGTCTTTG
TCGTGTTTACCTTACAATTTCATTGAAATTCATTTAATACATAAAGTATTAAAAAAATTAGAGTT
TCATTTTACTATACATTGAATTTTGTTTAGTTTATTAATAAAATCAGATAAGATGCTGAATGGAAT
TTCAAAAAGTTGTATGTAGAATTCTGAGTTTTTGAACAACATATATAACTCTCAGTTTTTCTTTT
TAAATAAACCTCAAGTCTCAAACCAAAGATTTTGAGCAACATATATAATATTAGAGTTAACTTT
GTTTAGTATTGATATTTGAAGGGGCCAAAATCAAACTTCACAGTTAAATATAGTGACAAAGGA
TAAAGCTTTCTCTTGGCCCTCAAATAAACACGTATATGACTTTGGGTCATCTTCCCCATCAAAG
AAAAAACTTGTGTTTTTTAAGCCTTTACTGATAAACCCATTACCTTATAAAGTGTATGTTTATTA
TCATAAACACAACTAAGAGGTGAGCCAAAGGCAAAGAGGTAATCAAGTTTTTGAAAACCTTA
TTTTCTTTATTTTTTTTTCAAAAAAGAATTCAAATCCGTGTCCAAATCCGTGTCCGGCCGTGTCC
AGTGGCGGAGCCAGGAAATGTAACTATGGGGGGCGGTGAGCAATGAGAAGAGAGAAATATT
TTTTATTTTCGTGTAACTTCTAAACAATCGGGTCGGGTCGGATTTAGTTAAGAGCAAAACACTC
TAATTCCAAGCAATTAACACTAATAAGAGGTGTAGGTGTCTATAAAATGAAACTTTGTTTATGT
GGGTAATATCAACCAGTTTACCATAAAAACTAGTCGCCTTATATTATCAATTACTTAGACGAGA
GTCCGAGACAAACAAAACATCGCTCAAATGTTTTATTGTATTTTTAACTTATCATAAAACCATA
ATAAATATCAATAGCATAACTAAAATCTTGGAAAAATAATTTATTATTAGAAAGCAAAGGAAA
CTTACTGTAAAGTGATTGTTAGTGGCCAATTGTATTACTACAACATTTTTTTGCTAACTTACTTAT
CAAACAATATGTACTGCTGGTACTATTTAAATAAGTAAACTACCTTTTGTATGTTAGTATTAGAT
AATGAAATGAATAATACACAGATCCAGCAGTCACAAGCCTAAACTCTTGGTCTCCACTTATCC
TCTTCCCCATCTTATCTTTCCCATATTTCACTCACCCCATAAAACAAAACCAGAAACCCATAAA
GAGAAGAAAAAAAGCCATTGCTAAGTTTGTTGAGTGGCAAAATTCAATCCAAGAGCACTTGT
TGTTGGCTTCCACTCACAAGTGAGGGCACTGGAAATTTTGAGCTGAGGGCACTTACTGTTTTA

ACACCATATTTGCATTTTTGTTCTTACTAGTTTACTGTATCTACAAGAACATGGTTGGGGGCGCT
TCCCCCCACTACCTTCTACATAGCTCCGCCCCTGGCCGTGTCCGACCCAAACAGCACGTGGAA
CACGGCACATGCGTGTCCGACACGTGTCCGAGCCGTGTCCGTGTCCGACACTGCCACGCCTT
ACTTGGAGCTGTGTCCGTGCTTCCTAGTATCCGATATATGTACTCATTTCTTTTTAGCTAATTTT
CGTTAAGGATCTTGCGAAATAGCTAGGCTAAAAATAAATTGATCAAGTTGTAGTACTTCGTCGC
CGTTCAAAAAAAAAAAAAAAAATTGTAGTACTTCGTATTTGATTTATTGATCACATATACAATG
ATGATGATAAACTTCCATTACAGACTGCAATATTTATGGGGAGC

>*CmbHLH30*

CTCTCTATAAATTACCTTTACTCTCTGTAAATTACCTCTTTCAATCGACTTTATTTATGACCAGAT
GAACATCTTTTTACATTTTAAACTTAACCCTAAATGCTATATATTGGATTTTTTTATTCTTTTTACG
TGTTTAACAAGCATATAAACACGCGTGTACTATTTGAAAGGGTTTTTAACTTAGAAAAAGACA
AAATTAAAGTATAATAAAAAAGCAGAAAGATATAGGACTTACATAAAAGATGTAATCAGATTT
TTTATGTGATCCCCATGCATTTTCATGGCCTCTACGTATTTTTTCGCATGTTAAAACCCTATTTGA
AAAGTAGCCCTTTTGTTGAACAACGGCTATTATACATGTGGGCTAATTAGTCGCTATCAATTATC
AAAGGAGGAAATAACTTATAGAAAAGTATCAATATAATGGACCATCCATGGAAGAAGAAATC
ATCAAACAAATACCACGGTTGAATTTCAATCTATGACAAAATACTCCTTCTGATGTCATCTTTC
TTGATATATGCACATGTGTTTATGCATATATATACACCAACCCTAAAAATTTCAACAGGGTTTCT
GTAAACAAAACTTATTAAAAATGGACTCCATTTTCAATCTTGAAGAAACAAAAAGATCTCGCT
TGCTTCGACAAATCATGGACTCCTTTGGTTTCACTTACATTTGCTTATGGTCTCACTTTTCTCATC
CTTCCAAGTACATCTCTCTCATTCAAGCTCAATTCTTTTTATATAGTATTCTTTGCAGTTCAAAA
AGAAATGTTTAATCATCTTTGATACCCATGCACAAATTGTAGTTGTTTGATCTTCATTGATGGAG
TATACATAGAAGGAAAGAATCATGCAAGCACATCATATGGGAGTCTTTCCGTGAGGTCATTTC
TTGACTACAAAAAGTCGGTGTTCTTGATTGATAATTATAGTGGGTAAGAACAATAAAAACACA
ATCTTTTTTCGTATAATGAGTATTACGAAAACTCAAATGTATGCCAGGGGTTTTAACTGAATAAT
GTTATGTTATTAGAGGAGTTCCAGCGTTTGCATTCATGCACAATATTACATACATGGAGCGGAA
TGAGTTGGAGCTTTTAAATTTGGCATCAAATTTTGCACAACTACAATTTTATCAAGTAATCACA
CTTCATTTATAATTAGTATTAGTTTAGTTTAATCCATGTTAAAGTTGAAGCTAATGTGTATCCCTA
TTTTATCTTGCAGGAAGCAGGGATTAAGGTATCCAATATATGTACTCAATTCGTTTTTAGCTAAA
AAATCAATTGATCAAGTTGTAGTACTTTGTATTTTATTTATTGATCACACATAAAATGATGATTAT
AAACTTCCATTACAGACTGCAATATTTATGGGGAGCAGCAATGGAGAGATTGAGCTTGGCATG
ACCAATGACTCCTCTCAAATAAGAAATGTAATTCACATCTTTCATATTGTGATCCTTTATTCTTC
GGTATTTATGTTTATTTCAGATTATATTAGTCTTATATAATTCGTTTAAATGGTTTAATTTTGGGTTT
TTTTTTTTAATTGTAGATTTTAAACTTTGAAATCGAGTTGAAGAAGTTGTTTCCAGGTGATTTCC
CTCAAGGAGTGCTTCCTCAACAACTTGAGCAAGCTCGGGCCTTATCTTCTTCATCATCTTTAAG
ATCATTATCAATGGACAACAGCATGGAGAACTCACCATTTCTCTTCAACATGCTTCATTCCACT
CCTTATATGTCCGAAATGTTTGCTCTCACAGAACCACATATGGAACAACAAGCACCAAATCAA
AAACCACAGTCAACAACAATATTGAGATCAGAAGATCCTCTGCGGCAAGCTTTACAACAAAT
TAGAACCTCTCATTCGATACCTTCAAGAGAACACGAAGAAGCTGCAATGACAAGAGCAATTC
TTGCAGTCATTACTTCTACTTCTTCTCCTACTCCTTCTTCTTCATTTCCATACCAGCCAACCCCCT
AGAGTTGCTAGTGCTTTTAAGAGACATGGATCAAGTAATTTAGGACCTATTAAACAACGAGTT
CCAAATCGACAAAATCTTCACAGAAGGTCACTTTCATTCTTCCGAAATCTAAGTGAGGCTCGT
GCTCAAAGAGATCAA

>*CmbHLH60*

AAAGACTTATCAACACCATCCAGAAGAATCATGCAGCCTGATCATTTACATTTATCAGACACG

CTTAGAGTATACTTCAAGATACTTGCTAAAGTATGTCGAAAGGATCTGGCTGAAGTTCTCAAA
GACGCTTAGGCGGGAGTAATTTGAGATAAGGCAAAACGGGAAAACCCTTGGGAAGATAGAG
ACTCATCGAGAGTAGGGAAACCAACTTACATGAGGTGTCAGCACAGTAAAGCAACCATAGCC
ACACGATTGCACGTGTACCTTACTTCCAGTTTGCTTCTAGTTTAGATATCATTGTTCTCTTGTAA
CTACTAACAGTGTATCTCCCTAGTTTATAAAGGGAGGTTAGTAGTAGTGATAAGCAATCGATTC
CACCACTAGAAACTTCCTTCTCTTCTAAGTGGGATCTTTCTCTTCATAACTTGAAGAGTAACAT
TTGTCATTACCGGGGATAGTATTCCGCAACTATCCCTCGTAAAGCCTTCACCTTAACTTCTAAG
TGAGACTAATACAGACGTAGTGTACAAATGGGTATTATTAGTTGACTCAGGTCAAGATTGTGTA
AACATGCTCTTATTTCTAATGAACATCGGTTATTTTCATTTGCTCAAACTCTTTTTCATACGTTAC
ATGGTTTTACACAAAAATATCGGACCAACAATCCAAGTTATTAGTCATCGGTTTCGTTTACCCA
ATAAACTCGAGAAAAGAGGGTAATTTAATCCAAACAACGATAATCCACAATCTACTTTTTAAA
GTTGATCAATTATAAAATTCTTTTCAGTTGATGTATCACTTAAAAAAAACTTTTGACTAGATAGA
TAACAAAGTTCTAAAATCGTACGTGATTACACTTTTAGATGTAGTTAAAACAAATTTTAACCTA
TTTAAAAACAATATCCTCCGGATTATTGTTTAAATCAAAAGTCCCAGCTTAAATTAGAATCATC
CCTTCCAAATCACTAATTAATTACCATTTCAAATCAAGTACATAAATAAATCTACTCAATCATAC
TAAATAAAGCTAAAAATAGGCCTAATTAATTATACTAAACATGAAAAATAAGAAAAGTGAAG
AGAGGGATTCGAAAGCACCGAGTTTGGGTAAGGAATGATGATGACGAAACAAATACTAAACT
AAAACCCAACCAAAGCTTGTAAATAACCCAAATGCTCCTTAACACCCAATAATCTTCGACTAA
TCTTTGCATGAATGAAACTTTATGTAAATTTAAGATGGTGAAATGAATAAAGTTCGCAACTTTA
TATTGTAGAAAGCTTAAAAACTTATAAAACTTGATTTAAGAACTATATCTTAAAATAAATTGCTT
AAAACTTTAATAAAAAACGTGATACAATTAAGTTTTAAAAATAAAAAGATGCAAGCTTTATAA
ATTTTAAAGACCAAAAAGGATGACTTTTATAAAAAGATTGAAACTTTATAATTCAAGTCACAA
ACATCAAAAGAAAATTTACATCATCTGCTCACAAATACTCCAACATAGTCACAAATACTCCAA
CATATACAAACACCTTATTCGTTGATAATACAAATATGTCACTTATGTGTGATAAAAGTTGTTTG
TAATCCAAACTATTTAGACACTAGACACTGATATTTTTGCATGTAATATCTTCATTAAAATGCAA
AAGAATCTTGTGAAATTAGGAAAGAGTAGTTATTAAACGGTTGGGATTAAAACTTGATGGTGG
TATTAGGAAACACCAGCCGCCTCAATTGAAACCATTTATCTGAACCTGCCTACTTCCACGTAG
GATCCTGACACTTATCACTAGCCAGCTAGGTTCCTTGTATGTAGTATTGTAGTTTCCTTAAACAT
CCATGGGCAATTTAGGTATTTTAATATTCTTCACAAGAATCTCATGCTTCTATATAAAAGCTACA
CTTTTCCCACTTCCCCTCTTGCCATTTTCACAAATAGATAACAACAAACAAAACACATTTGTTG
AAACATACGAATATTTGTTCAAATCCGTTAAATTGCGTACATATCTGTCACTAGAGGTACAAAT
TTGTACGATTCTGATA

Table S2 Number of bHLH TF in each subfamily of chrysanthemum and *Arabidopsis.*

Subgroup	Chrysanthemum	*Arabidopsis*	Rate (Chrysanthemum/*Arabidopsis*)
Ia	3	10	0.30
Ib	0	10	0.00
II	2	4	0.50
IIIa+c	3	5	0.60
IIIb	1	4	0.25
IIIe+d+f	5	12	0.42
IVa	8	4	2.00
IVb+c	4	6	0.67
IVd	4	1	4.00
Va	0	3	0.00
Vb	6	5	1.20
VIIa(1)	0	5	0.00
VIIa(2)	2	8	0.25
VIIa(3)+b	0	5	0.00
VIIIa	3	3	1.00
VIIIb	2	6	0.33
VIIIc	0	6	0.00
IX	5	5	1.00
X	4	5	0.80
XI	2	5	0.40
XII	10	17	0.59
XIII	2	3	0.67
XIV	0	5	0.00
XV	0	3	0.00
Orphans	5	16	0.31
Total	71	156	0.46

Table S3 Proportion of conserved amino acids at each point of CmbHLH protein.

Amino acid location	Conserved amino acids >50%	Ratio (%)
5	His，H	55
8	Ala，A	59
9	Glu，E	77
10	Arg，R	68
12	Arg，R	77
13	Arg，R	92
14	Glu，E	52
23	Leu，L	83
26	Leu，L	61
27	Val，V	51
28	Pro，P	76
35	Lys，K	56
37	Asp，D	65
38	Lys，K	59
39	Ala，A	79
40	Ser，S	52
42	Leu，L	82
45	Ala，A	56
46	Ile，I	63
48	Tyr，Y	70
49	Ile，I	54
50	Lys，K	52
52	Leu，L	92
56	Val，V	55
59	Leu，L	75

Table S4 Selection criteria for differential genes.

Gene ID (name)	Cm_A3 vs Cm_CK0		Cm_A5 vs Cm_CK0	
	log2FC	Padj	log2FC	Padj
c60363_g1(*CmbHLH16*)	3.9	8.29E-12	3.7	4.40E-16
c61229_g1(*CmbHLH18*)	2.4	0.00023616	1.6	0.0050347
c93607_g1(*CmbHLH28*)	3.5	4.53E-19	2.7	2.4474E-23
c93607_g2(*CmbHLH30*)	4.1	3.06E-14	3.7	1.1979E-20
c77068_g1(*CmbHLH60*)	3.8	2.27E-43	2.7	1.3631E-21

Note: Cm_A3 vs Cm_CK0 represents the ratio of the expression of this gene at day 3 of inoculation to that at day 0 of inoculation by *Alternaria* sp.; Cm_A5 vs Cm_CK0 represents the ratio of the expression of this gene at day 5 of inoculation to that at day 0 of inoculation by *Alternaria* sp. The screening criterion for differential genes is the default value of log2FC is greater than 1 and Padj less than or equal to 0.05.

Table S5 Relative activity of leaf cells of WT and transgenic chrysanthemum.

Lines	A_{600nm}	Relative cell activity/ %
WT	$1.237±0.178^{a}$	54.93^{a}
35S::*CmbHLH18–5–1*	$0.525±0.067^{b}$	80.86^{b}
35S::*CmbHLH18–32–9*	$0.370±0.040^{bc}$	86.53^{bc}
35S::*CmbHLH18–14–4*	$0.276±0.008^{c}$	89.95^{c}

Note: The different letters signified significant differences at $p < 0.05$ by one-way ANOVA.

Table S6 Primers used in this study.

Primer codes	Forword primer (5′-3′)	Reverse primer (5′-3′)	Purpose
qCmbHLH16	CTTCAAATGACACCAAC GAGGAT	GAGGCGGGCGGAATACA	For RT-qPCR in Chrysanthemum.
qCmbHLH18	GGCTGCAATTCAAAGGA TGTTC	GTCATGCAGAAGTCGTCCG CC	
qCmbHLH28	TTCTGAGATGGCGGTAG	CGTGGCACATCCTCTTT	
qCmbHLH30	TGTAGTTGGCATCACCG	CAAATCCACCAGTATCACA T	

qCmbHLH60	CTTCTGATGGCGAGTTT	GGTTTGTCCACCTATGTTAG	
CiUBI	AGCTGAGCAGACTCCCGATG	AGGCGAATCATCAGTACCAAGT	For RT-qPCR.
CmbHLH18	GGTGTCCAAATCATCCCAGAA	GGATCGGACAAGCAACCGTA	For isolation and transformation of *CmbHLH18* into vector.
Super1300	GCCATTTCGCCTTTTCAG	TGATAATCATCGCAAGACCG	
CmbHLH18-V	CTAGTCTAGAATGGGAAAGGTATCGAACACGATT	CGGGGTACCAAGAACTTGTTGCAGATTCTTCGA	
CmbHLH18-At	TGAAGTGCAAATGTCAGGGAA	TACTTCCTACTCCCACCAA	For identification of transgenic *Arabidopsis.*
AtActin	CTAAGCTCTCAAGATCAAAGGC	AACATTGCAAAGAGTTTCAAGG	For RT-qPCR in *Arabidopsis.*
AtSOD	TGGAACTGCCACCTTCACAAT	GATGCCGCAAGCAACACG	
AtCAT	ACGCCGATTTGCGAGATACA	CAGACAGGAGCACCCGAGTTT	
AtPAL	AAGCCTGAGTTCACCGATCATC	TACGAGCTTCCGTCGAGGAT	
AtPOD	TGCGAGAGACGGTATTGTCA	TCTCCCAAGTAGCTCCCTCT	
AtCHT	CGGAAACTGGAAACCTACA	CAGTTGAAGGGTTGAAAGG	
AtGLU	GCAATGCAGAACATCGAGAA	TCATCCCTGAACCTTCCTTG	
